# Complete chloroplast genome sequence of *Adenostemma lavenia* (Asteraceae) and phylogenetic analysis with related species

**DOI:** 10.1080/23802359.2021.1944369

**Published:** 2021-07-06

**Authors:** Mingze Xia, Yan Li

**Affiliations:** a Key Laboratory of Adaptation and Evolution of Plateau Biota, Northwest Institute of Plateau Biology, Chinese Academy of Sciences, Xining, China; b University of Chinese Academy of Sciences, Beijing, China; c School of Pharmacy, Weifang Medical University, Weifang, China

**Keywords:** *Adenostemma lavenia*, chloroplast genome, phylogenetic analysis

## Abstract

*Adenostemma lavenia* is a perennial medical herb in the family Asteraceae. Here, we sequenced and analyzed the complete chloroplast genome of *A. lavenia*. The complete chloroplast genome size is 150,063 bp with a GC content of 37.63%. The *A. lavenia* chloroplast genome is a typical quadripartite structure, including a large single-copy region (LSC) of 82,017 bp and a small single-copy region (SSC) of 18,142 bp separated by a pair of inverted repeats (IRs) of 24,952 bp each. A total of 114 unique genes, including 29 tRNA genes, four rRNA genes, and 81 protein-coding genes were found in the chloroplast genome. Phylogenetic analysis revealed that *A. lavenia* is more closely related with *Chromolaena odorata*.

Chloroplast genomes are closed circular DNA molecules in most angiosperms, which show a typically quadripartite structure of a large single-copy (LSC) region and a small single-copy (SSC) region separating by a pair of inverted repeats (IRs) (Palmer 1985). Chloroplast genomes are useful in the development of DNA barcodes for identification (Daniell et al. 2016), and can improve the resolution of phylogenetic relationships in large and complex plant lineages (Doorduin et al. 2011; Dong et al. 2018).

Comprising *ca*. 25,000–35,000 species in at least 1600 genus with the distribution of all continents except Antarctica, Asteraceae is considered the largest flowering plant family (Funk et al. 2009). *Adenostemma lavenia* (L.) O. Kuntze 1891, a perennial herb of the family Asteraceae, is widely distributed in tropical and temperate region of Asia and Pacific Islands (Cheng et al. 1979; Irmanida et al. 2020). In traditionally, the whole plant has been used as a medical herb to treat bronchitis, tonsillitis, pneumonia, lung congestion, hepatitis, fever and malaria (Cheng et al. 1979; Yang et al. 2007; Irmanida et al. 2020). Although this species possesses a wide distribution range and a large number of biological resources (Cheng et al. 1979), the related study of molecular biology is rare.

Fresh leaves of *A. lavenia* was sampled from Enshi City, Hubei Province (Geographic coordinates 30°28′N, 109°49′E) and quickly dried in silica gel. A specimen and DNA were deposited at the Herbarium of Weifang Medical University (Yan Li and liyan715@mails.ucas.ac.cn) under the voucher accession number LY2020001. Total genomic DNA was extracted from approximately 10 mg of silica-dried leaf tissue by the modification of CTAB method (Doyle and Doyle 1987). The extracted DNAs of all the individuals were then sent to Novogene (Beijing, China) for genomic library construction and Illumina sequencing. Paired‐end reads of 2 × 150 bp for all samples were generated in a single lane on an Illumina HiSeq2500 sequencer (San Diego, CA, USA). We sequenced 10.21 Gb raw data, then used Trimmomatic v. 0.33 (Bolger et al. 2014) to control the quality of reads and obtained 10.14 Gb clean data. The chloroplast genome was assembled *de novo* by using GetOrganelle with the specific parameters (the assembling type: embplant_pt; the maximum number of extending rounds: 15; the K-mer length: 21, 45, 65, 85 and 105) (Jin et al. 2020) and visualized assembly result in bandage v. 0.8.1 (Wick et al. 2015). Annotation was performed through the online program GeSeq (http://chlorobox.mpimp-golm.mpg.de/geseq.html) (Tillich et al. 2017) and then manually adjusted for start/stop codons and intron/exon borders in SEQUIN Version 15.50 (https://www.ncbi.nlm.nih.gov/Sequin/) after BLAST searches. The chloroplast genome composition of *A. lavenia* was displayed using Chloroplot (Zheng et al. 2020). Sequence length of complete genomes and junction sites of LSC, IR, and SSC regions among *A. lavenia* and six other Asteraceae species were compared by using the program IRscope (Amiryousefi et al. 2018).

The complete genome size of *A. lavenia* is 150,063 bp in length, containing the large single-copy (LSC, 82,017), small single-copy (SSC, 18,142) and two inverted repeat (IR, 24,952) regions. Overall GC contents of chloroplast genomes were 38%. A total of 114 unique genes, including 29 tRNA genes, four rRNA genes, and 81 protein-coding genes were found in chloroplast genomes. In addition, comparative analysis revealed that the chloroplast genome size of *A. lavenia* is the smallest among the compared species of Asteraceae, but the organization and gene content is highly similar among species except *H. tuberosus*. Phylogenetic analysis was performed on the chloroplast genomes sequences of 14 species (including 7 species of Eupatorieae, 5 species of Heliantheae, and 2 species as outgroup of Menyanthaceae). Protein sequences of each species were selected as dataset for phylogenetics analysis, and then compared with MAFFT (Katoh and Standley 2013). Bayesian inference (BI) was performed by PhyloSuite v1.2.2 (Zhang et al. 2020). The Monte Carlo Markov chains (MCMC, one cold chain and three hot chains) analysis was run for 1,000,000 generations and trees were sampled every 1000 generations. The result showed that *A. lavenia* is closely related with *Chromolaena odorata*, *Ageratum conyzoides* and *Praxelis clematidea*, and all selected species of Eupatorieae and Heliantheae form a well-supported clade, respectively (Figure 1), supporting phylogenetic relationships of Asteraceae (Fu et al. 2016). This study will be helpful for future studies on chloroplast genomes and phylogeny of *Adenostemma.*

**Figure 1.**
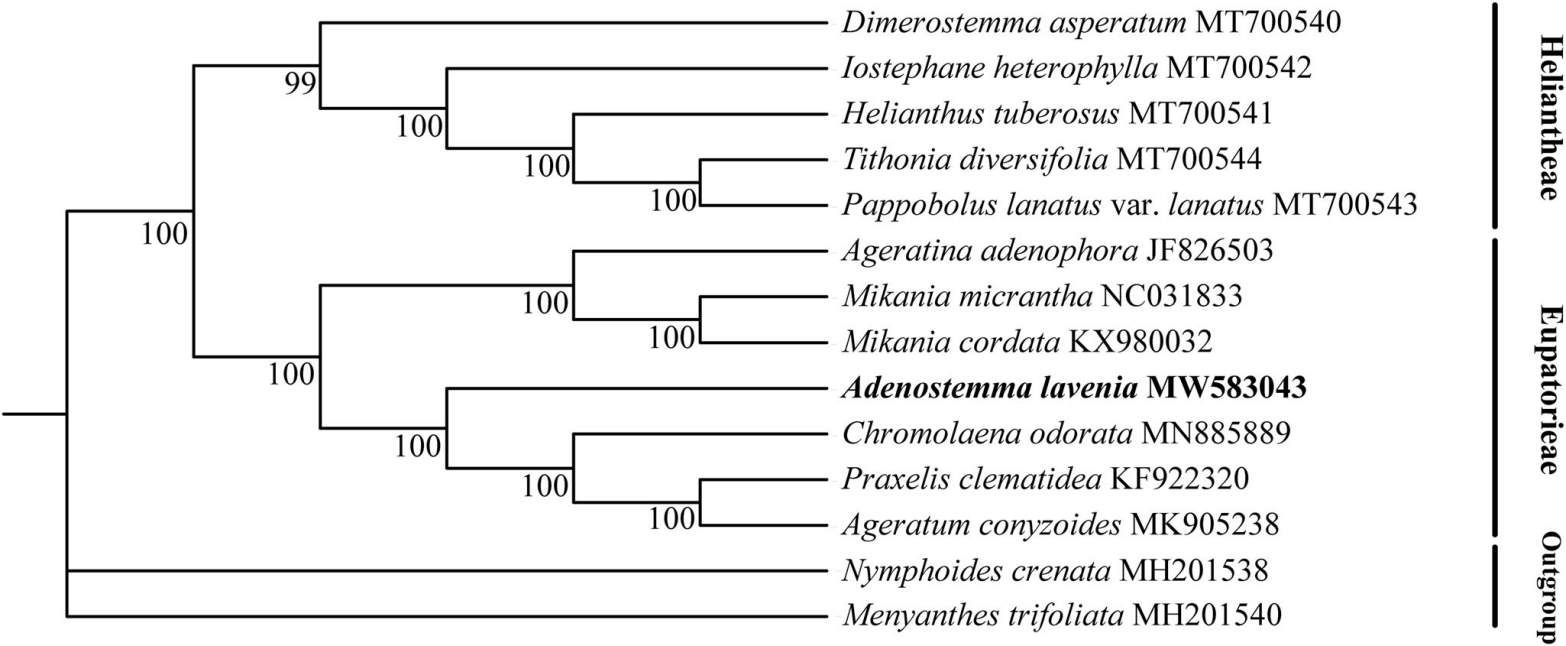
Bayesian inference tree based on the protein sequences of 14 chloroplast genomes. Numbers on the branches are posterior probabilities.

## Data Availability

The genome sequence data that support the findings of this study are openly available in GenBank of NCBI (https://www.ncbi.nlm.nih.gov/) under the accession no. MW583043. The associated BioProject, SRA, and Bio-Sample numbers are PRJNA732125, SRR14626653, and SAMN19312750, respectively.
